# The Effectiveness of Health Animations in Audiences With Different Health Literacy Levels: An Experimental Study

**DOI:** 10.2196/jmir.3979

**Published:** 2015-01-13

**Authors:** Corine S Meppelink, Julia CM van Weert, Carola J Haven, Edith G Smit

**Affiliations:** ^1^Amsterdam School of Communication Research/ASCoRDepartment of Communication ScienceUniversity of AmsterdamAmsterdamNetherlands; ^2^Clinical Skills Training CenterUniversity Medical Center GroningenGroningenNetherlands

**Keywords:** health literacy, animation, medical illustration, reading, audiovisual media, cancer screening, colorectal cancer, prevention, memory, attitudes

## Abstract

**Background:**

Processing Web-based health information can be difficult, especially for people with low health literacy. Presenting health information in an audiovisual format, such as animation, is expected to improve understanding among low health literate audiences.

**Objective:**

The aim of this paper is to investigate what features of spoken health animations improve information recall and attitudes and whether there are differences between health literacy groups.

**Methods:**

We conducted an online experiment among 231 participants aged 55 years or older with either low or high health literacy. A 2 (spoken vs written text) x 2 (illustration vs animation) design was used. Participants were randomly exposed to one of the four experimental messages, all providing the same information on colorectal cancer screening.

**Results:**

The results showed that, among people with low health literacy, spoken messages about colorectal cancer screening improved recall (*P*=.03) and attitudes (*P*=.02) compared to written messages. Animations alone did not improve recall, but when combined with spoken text, they significantly improved recall in this group (*P*=.02). When exposed to spoken animations, people with low health literacy recalled the same amount of information as their high health literate counterparts (*P*=.12), whereas in all other conditions people with high health literacy recalled more information compared to low health literate individuals. For people with low health literacy, positive attitudes mediated the relationship between spoken text and the intention to have a colorectal cancer screening (*b*=.12; 95% CI 0.02-0.25).

**Conclusions:**

We conclude that spoken animation is the best way to communicate complex health information to people with low health literacy. This format can even bridge the information processing gap between audiences with low and high health literacy as the recall differences between the two groups are eliminated. As animations do not negatively influence high health literate audiences, it is concluded that information adapted to audiences with low health literacy suits people with high health literacy as well.

## Introduction

### Background

Web-based information about health and disease prevention is widely available. In 2013, the majority of the people living in the United States and The Netherlands used the Internet to find health-related information [1,2] and many people consider the Internet a valuable tool for finding health information [3]. However, a significant portion of the potential audience fails to understand Web-based health materials due to low health literacy [4]. This is problematic because health information could be valuable for this group. People with low health literacy are, for example, more often chronically ill and less likely to use preventive health services, such as cancer screening, compared to people with high health literacy [5]. To reduce health disparities in society, there is a need for health information that is easily understood and appreciated by people with low health literacy and that is not rejected by people with higher health literacy levels.

The rise of online communication has offered many new possibilities to make health communication more attractive, especially for people with low health literacy. On the Internet, information can be presented in various delivery modes such as videos or animations. A study on tailored feedback, delivered by text or video, showed that video computer tailoring was more effective than text computer tailoring in realizing smoking cessation [6]. A recent literature review, however, concluded that print and audiovisual information often perform equally well [7]. The authors argue that audiovisual messages are promising but that there is a need for well-designed experiments comparing different formats while keeping the content the same. The different message features make it difficult to compare both formats and to draw conclusions about the effective elements. For example, a video presents both visual and auditory information, which is assumed to improve information processing [8], but people with low health literacy can also suffer from paying too much attention to irrelevant details [9]. Therefore, animations consisting of simple line drawings could be preferred over realistic videos that often capture many details.

The aim of our study is to investigate how text modality (written vs spoken) and visual format (illustrations vs animations) influence health information recall and attitudes and whether this differs between people with different health literacy. We will focus on health animations in which the textual information is clearly depicted. An animation is defined as “a simulated motion picture depicting movement of drawn (or simulated) objects” (p 88) [8].

This study adds to the literature in the following ways. First, we move beyond the comparison of different media formats and try to identify the specific message features that affect processing by using an experiment. Most of the studies conducted in relation to health literacy are cross-sectional and do not test possible mechanisms [10]. Furthermore, our study responds to the need for effective population-level health literacy interventions. Intervention studies conducted in non-clinical settings, particularly with regard to communicable diseases, are scarce in Europe [11]. The topic addressed in this study is colorectal cancer screening. People with low health literacy participate less in cancer screening [12], which highlights the relevance of studying the effectiveness of cancer screening messages in this group. Colorectal cancer screening is particularly relevant to older people, as all people between 55 and 75 years are invited to have this screening in the Netherlands [13].

### Text Modality: Visual (Written) Versus Auditory (Spoken)

Animations and written information fundamentally differ by text modality, or the way in which text is presented. Textual information in animations is often spoken, whereas leaflets or websites consist of written text. The cognitive theory of multimedia learning describes how people learn from words and pictures [14]. This theory is based on a dual-channel assumption, suggesting that people have separate channels to process visual and auditory information [14,15]. Both channels are expected to have their own limited processing capacity. This means that information presented in both modes (visual and auditory) is stored in memory better than information presented in a single mode. In written messages, both text and pictures are visual and processed by the eyes. Animations, in contrast, consist of auditory text and visual pictures. By using two modes, animations are expected to decrease the likelihood that the receiver experiences cognitive overload. Cognitive overload hinders information processing. According to the limited capacity model of motivated, mediated message processing [16], a message will be better processed, stored in memory, and retrieved at a later moment when people have sufficient cognitive capacity available. The final processing stage, information retrieval, is indicated by information recall. Based on this, it is hypothesized that: Health messages with spoken text (vs written text) improve information recall (H1).

Information recall is not the only important outcome in health communication. Next to optimal knowledge, positive attitudes are also required for informed participation in cancer screening [17]. Text modality could be expected to influence people’s attitudes toward a message by means of processing ease. Information addressing both eyes and ears (ie, audiovisual) could be easier to process than information addressing a single mode (eg, written). Literature on processing fluency subsequently states that the ease with which people process stimuli affects people’s preference for those stimuli [18]. Thus, people could be expected to have more positive attitudes toward messages that are easily processed compared to messages that are difficult to process. This idea has been confirmed in a study on websites, which showed that websites that include both visual and auditory information were associated with more positive and enduring attitudes toward the website compared to websites that included only visual information [19]. It could be expected that messages based on visual and auditory information positively influence people’s attitudes toward the message. This leads to our second hypothesis stating: Health messages with spoken text (vs written text) result in positive attitudes to the message (H2).

### Health Literacy

Health literacy refers to “the degree to which individuals can obtain, process, understand, and communicate about health-related information needed to make informed health decisions” (p 16) [20]. It is a broad concept that is still evolving [20]. Health literacy is closely related to functional literacy [21], which means that people with low health literacy often have reading problems as well. For this reason, spoken messages could be particularly effective for audiences with low health literacy because no reading is required [22]. Additionally, groups with low health literacy often lack the health-related background knowledge that is required to understand information [23]. Low health literates are, therefore, easily at risk of cognitive overload when presented with health-related information [24]. Reduction of cognitive load by using message features that enable processing could, therefore, be especially salient for people with low health literacy. For this reason, our third hypothesis states: The positive effect of spoken text (vs written text) on recall and attitude to the message only exists among people with low health literacy (H3).

### Visual Format: Illustration Versus Animation

The other feature that distinguishes animations from written texts is moving visuals. A meta-analysis on the effectiveness of animations versus illustrations showed that animations generally result in better learning outcomes [25]. The authors state that an animation can provide an external model for a mental representation. As learning and understanding encompasses the creation of an adequate mental representation [14,26], animations will be better able to support this process compared to illustrations. This will apply particularly to audience groups that have limited knowledge available to build such mental representations themselves, such as people with low health literacy.

Based on the above reasoning, it could be expected that animated visual content improves information processing compared to illustrations. However, this will not always be the case. Movement in animations requires more visual attention from the viewer compared to still illustrations. It is suggested that, compared to illustrations, animations require a higher level of awareness from the receiver due to the ongoing changes in the visual information [27]. This may increase the cognitive capacity that people need to properly process the information. Receivers are expected to handle this increased cognitive load better when they are able to listen to the text rather than reading it. Thus, to reduce cognitive load, the textual information in animations has to be spoken and not written, particularly for people with low health literacy, as they are more likely to experience cognitive overload. Therefore, it is expected that animations (vs illustrations) positively affect recall, but only if the text is spoken (H4a). This interaction effect will only exist among people with low health literacy (H4b).

Next, other than improving recall, moving visuals can also positively affect attitudes toward the messages. Most likely, it is vividness that makes an animated advertisement more appealing to the audience compared to an illustration [28]. Due to the movement of animations, people will perceive them as more emotionally interesting and imagery provoking. A study of online advertising revealed that people had more positive attitudes toward animated advertisements compared to motionless ones [28]. However, the positive influence of moving images on attitudes is only expected in the case of spoken text messages. As animated visuals and written text are both processed by the eyes, people have to divide their visual attention between the text and the pictures. Moving objects automatically capture the visual attention of the viewer [29]. Thus, a combination of animation and written text increases cognitive load, resulting in less fluent processing. This could negatively affect attitudes toward the message. Based on this evidence, it is expected that animations (vs illustrations) positively influence attitudes to the message, but only if the text is spoken (H5).

### Sequential Message Effects

In addition to knowledge improvement, information about cancer screening often aims to convince people about the screening’s benefits. Ideally, screening participation should be based on informed decisions. This means that people need to be properly informed about the screening’s benefits and disadvantages and they also need to hold attitudes toward the behavior that are congruent with the actual behavior [17]. From a communication perspective, however, it can be expected that people’s evaluation of the message affects their attitudes toward the behavior. If the features of a message about colorectal cancer enhance information processing, experienced fluency will induce a positive attitude toward the message [18]. For example, positive attitudes toward the message can be transferred to behavioral attitudes, which is called the spill-over effect. Spill-over effects have been found in other fields of communication where positive attitudes toward an advertisement or game positively affect brand attitudes [30]. Thus, a positive attitude toward a cancer screening message could improve attitudes toward the screening itself.

According to the theory of planned behavior [31], attitudes toward the behavior affect behavioral intention. This relationship has often been confirmed in health research [32,33], suggesting that someone with a positive attitude toward cancer screening is likely to intend to screen as well. In concurrence with the preceding hypotheses, it is expected that this sequence of message effects induced by message format primarily exists in people with low health literacy. Therefore, our sixth hypothesis refers only to this group. It is expected that among people with low health literacy, spoken text (vs written text) improves the intention to screen for cancer. This relationship is mediated by both the attitude toward the message and the attitude toward the behavior (H6).

## Methods

### Design and Participants

A 2 (text format: written vs spoken) by 2 (visual format: illustration vs. animation) between-subjects design was used. Ethical approval of this study was provided by the Amsterdam School of Communication Research (2013-CW-5). Participants aged 55 years or older were randomly selected from a large respondent pool by the ISO-certified market research company PanelClix. A minimum age of 55 years was required due to the topic of the experimental messages: colorectal cancer screening. At the time of data collection, a national screening program on colorectal cancer was planned in the Netherlands, but the public had not been informed yet. Therefore, limited prior knowledge was expected. We nevertheless measured prior knowledge to control for its potential influence. An invitation was sent by email to 1295 individuals in November 2013, of which 397 unique participants started the survey (participation rate 30.66%). Uniqueness of participants was determined by the “pid-code” (this is an anonymous individual code assigned to participants by the research company). Two participants filled out the survey twice, indicated by identical pid-codes in the dataset. In both cases, the second entry was excluded from the analysis.

A stratified sample was created in which gender, different age groups (55-64 years, 65-74 years, ≥75 years), and high versus low education levels were equally represented. Low education level ranged from no education to the lower levels of secondary school (“VMBO”), whereas a high education level represented higher education or a university degree. We excluded the middle education group because PanelClix was not able to stratify the sample on health literacy, but it was possible to sample participants based on education level. As health literacy and education level are related, we decided to include only people with low or high education to make sure that enough low and high health literates were included in the sample. Most strata were properly filled (at least 20 participants), with the exception of highly educated participants over the age of 75 years. This could be because a higher education level is quite rare among people of this age, especially among women.

**Figure 1:**
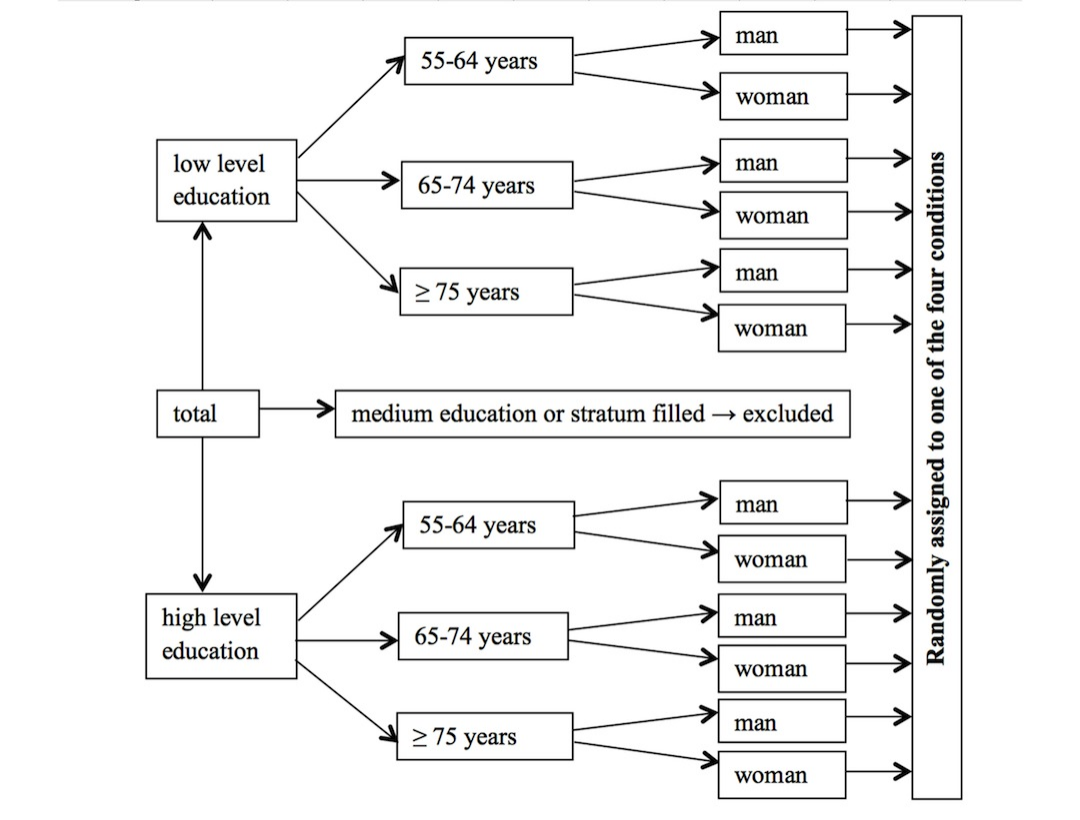
Flow chart of the stratification procedure.

Of the 397 people who viewed the first page of the survey, 353 (88.9%) continued after the informed consent page. After stratification, 250 participants (70.8%) were eligible to participate and 103 individuals (29.2%) were excluded because either their education level did not meet our inclusion criteria or the stratum to which they belonged was already full. Of the eligible participants, 16 people (6.4%) quit during the experiment, and three (1.2%) were excluded because they had not been exposed to any stimulus due to a technical issue. The mean age of the 231 participants who reliably completed the entire questionnaire was 68.22 years (SD 8.67, range 55-99) and 121 (52.4%) were male). The flow chart in Figure 1 provides an overview of the stratification procedure. Due to the stratification, participant’s gender, age, and education level were equally distributed over the four experimental conditions. Before the survey was sent to the participants, it was pre-tested several times among people of the target population who were not in the final sample. During these pretests, the duration and usability of the questionnaire was tested.

### Procedure

At the beginning of the questionnaire, participants were informed about the topic of the study, their anonymity and right to withdraw their data within 24 hours, the survey length, and contact details of the researchers. Subsequently, participants gave informed consent and answered the stratification questions about gender, age, and education level. If the participant fit in one of the strata, the questionnaire continued by asking for the participant’s professional medical background, knowledge about medicine in general, colorectal cancer, and colorectal cancer screening. Then, within each stratum, people were randomly assigned to one of the four experimental messages. All of the messages were self-paced and consisted of 15 separate webpages. Participants clicked a button to continue to the next page; returning to the previous page was not possible. The audio text and the animation started automatically and all parts could be replayed. We purposely provided the participants with the opportunity to replay the message as this enabled us to rule out pacing differences that would otherwise exist between the written and spoken/animated conditions. In the audio conditions, participants were clearly instructed to switch on their speakers or use headphones. They were also exposed to a test question, the sound of a ringing telephone, which was played to see if participants could identify the sound. After the experimental messages, attitude toward the message, information recall, attitude toward the behavior (screening), behavioral intention (intention to screen), and health literacy were measured. Participants were rewarded by receiving credit points from the research company. People could not miss any of the questions due to forced response settings and all responses were automatically stored into a database.

### Experimental Stimuli

**Figure 2:**
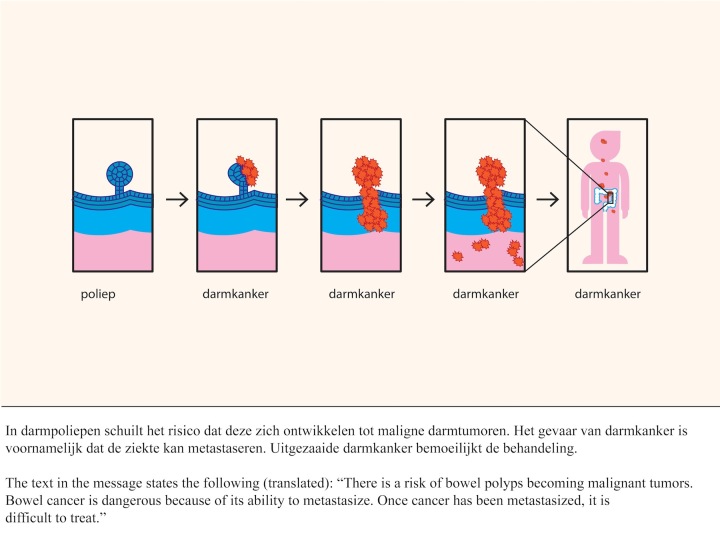
Example of the static picture and written text.

The experimental messages were about colorectal cancer screening, in which the following topics were discussed: the risks of colorectal cancer, the development of the disease, why early detection is beneficial, the procedure of the test (fecal occult blood test), and the possible test outcomes. Four experimental messages were created (450 words) based on information that was provided by the screening organization. These messages were complex (ie, written at C1 level in the Common European Framework of Reference for Languages). An extensive description of the development of the messages is provided elsewhere [34]. In the two audio conditions, the text was narrated by a professional Dutch radio news presenter. The simple, non-detailed illustrations were created for the purpose of this study and supported the text. Research has shown that simple drawings are comprehended better than more naturalistic drawings or photographs [9]. In the animated conditions, the illustrations were replaced by animations. Figure 2 shows an example of the illustration and written text. First, a healthy bowel polyp is depicted, followed by a polyp that has malign cells. The animated version shows a healthy polyp turning malignant (see Multimedia Appendix 1).

### Measures

#### Health Literacy

Health literacy was measured using the Short Assessment of Health Literacy in Dutch (SAHL-D) [35], which consists of 33 words related to health and health care, such as obesity, ventricle, and palliative. We used only the comprehension test of the SAHL-D and not the word recognition test because the first one is more relevant in the context of our study. When exposed to mediated health information, people should not necessarily be able to correctly read this aloud. It is more important to examine whether people understand the information. For each word, people were prompted to select the correct meaning out of three multiple choice options. Each correct answer received 1 point. If the incorrect meaning was selected, or people indicated that they did not know the meaning of the word, no points were awarded. Consequently, health literacy scores ranged from 0 to 33 (mean 23.20, SD 7.45).

#### Recall of Information

Information recall was measured with an adapted version of the Netherlands Patient Information Recall Questionnaire [36]. Participants answered 14 open-ended recall questions about the content of the messages by typing the responses into a text box. Based on a predefined codebook, the responses were scored, and each answer was marked 0 (false), 1 (partly good), or 2 (good). Consequently, total recall scores could range between 0 and 28 (mean 12.81, SD 5.90). Intercoder reliability was calculated for 19.0% (44/231) of the responses, coded by the first author and then a second coder who was not one of the authors, and appeared to be good: Cohen’s kappa=.90 (range 0.51-1.00)*.*

#### Attitudes Toward the Message

Nine items on a 7-point semantic differential were used to measure attitudes toward the message. The items were based on a measure for attitudes toward the information [37] and a Website Satisfaction Scale [38]. The items were presented in a randomized order to the participants. Participants evaluated the message using the following anchor points: provided bad feelings/good feelings, unpleasant/pleasant, not interesting/interesting, not informative/informative, not reassuring/reassuring, bad/good, not creative/creative, not appealing/appealing, and ugly/beautiful. The scale was reliable (α=.94, mean 5.95, SD 0.98).

#### Attitudes Toward the Behavior

Seven items, presented in a randomized order, were used to measure attitudes toward the behavior [39]. Participants evaluated colorectal cancer screening on a 7-point semantic differential scale, ranging from 1 (negative) to 7 (positive). The following anchor points we used: negative/positive, bad/good, undesirable/desirable, useless/useful, unimportant/important, unpleasant/pleasant, and not reassuring/reassuring (α=.93, mean 6.11, SD 0.97).

#### Behavioral Intention

Intention to participate in colorectal cancer screenings was measured with one item on a 7-point scale. People responded to the following statement: “If I am invited to participate in colorectal cancer screening, I will…” Answer options ranged from 1=definitely not participate to 7= definitely participate (mean 6.12, SD 0.97).

#### Control Variables

Participants’ knowledge was measured as a control variable using three items on a 7-point Likert scale (1=no knowledge, 7=much knowledge). The items referred to general medical knowledge, colorectal cancer knowledge, and knowledge of colorectal cancer screening (see Table 1 for means and standard deviations). People also indicated whether they had a professional medical background or not (ie, medical, nursing, or paramedical). Analysis of variance showed no differences between conditions in participants’ knowledge of medicine in general (*F*_3,227_=1.36, *P*=.26), knowledge of colorectal cancer (*F*_3,227_=1.78, *P*=.15), and knowledge of colorectal cancer screening (*F*_3,227_=0.99, *P*=.40). The groups were also found to be similar with respect to the participant’s professional background in medicine (*χ* ^*2*^_*3*_=4.08, *P*=.25).

### Statistical Analysis

To investigate the influence of text modality, visual format, and health literacy on information recall, attitudes, and intention, a multivariate analysis of variance (MANOVA) was conducted using SPSS 20. Health literacy scores of 24 and below were labeled as “low health literacy” and scores of 25 or higher were labeled as “high health literacy”. To reduce false positives (ie, people incorrectly categorized as low health literate), we used a cut-off point that is slightly lower than the optimal cut-off scores based on the full SAHL-D [35]. The cut-off point corresponds to the sample median (25).

PROCESS (model 6, 10,000 bootstrapped samples) was used to test the indirect effect of text modality on the intention to screen through both the attitudes toward the message and the attitudes toward the screening. PROCESS is a macro for SPSS [40] that uses bootstrapping to estimate 95% bias corrected bootstrap confidence intervals for total and specific indirect effects. Due to intention to screen being negatively skewed (skewness=−1.86, SE 0.16), this measure was first reversed to create a positive skew [41]. The square root values were subsequently re-reversed and used in the analysis. The mediation hypothesis concerned people with low health literacy. Therefore, only people who belonged to this group (n=108) were included in this analysis.

## Results

### Study Population

Table 1 provides an overview of participant characteristics.

**Table 1:** Overview of participant background characteristics (n=231).

Characteristic		n (%)	mean (SD)
**Gender**			
	Male	121 (52.4)	
	Female	110 (47.6)	
Age, years^a^			68.22 (8.63)
**Education level**			
	Low	123 (53.2)	
	High	108 (46.8)	
**Medical background**			
	Medical	1 (0.4)	
	Paramedical	9 (3.9)	
	Nursing	17 (7.4)	
	None	204 (88.3)	
**Prior knowledge** ^b^			
	Medical knowledge in general		2.92 (1.44)
	Knowledge of colorectal cancer		2.31 (1.38)
	Knowledge of colorectal cancer screening		2.53 (1.61)
**Health literacy** ^c^			
	Low (SAHL-D^d^score ≤24)	108 (46.8)	
	High (SAHL-D score ≥25)	123 (53.2)	

^a^Age ranges from 55 to 99 years.

^b^Prior knowledge scores range from 1 to 7, with higher scores indicating more knowledge.

^c^Health literacy ranges between 0 and 33.

^d^SAHL-D: Short Assessment of Health Literacy in Dutch.

### Effects of Text Modality and Visual Format in Different Health Literacy Groups

A main effect was found for text modality on information recall (*F*_1,223_=5.43, *P*=.02, η_p_^2^=.02). The means presented in Table 2 show that spoken messages were recalled better than written messages. Spoken messages also resulted in more positive attitudes toward the message (*F*_1,223_=7.90, *P*=.01, η_p_^2^=.03), supporting H1 and H2. Simple effect analysis revealed that the superiority of the spoken text modality on recall and attitudes to the message existed only in the low health literacy group and was not found in people with high health literacy. This finding supports H3.

The fourth hypothesis predicted a positive effect of animations (vs illustrations) on information recall. An interaction was expected because this positive effect was predicted only in spoken messages (vs written messages). No interaction was observed between Text Modality and Visual Format on information recall (*F*_1,223_=1.49, *P*=.22, η_p_^2^=.01), rejecting H4a. However, as predicted by H4b, a three-way interaction was found for Text Modality, Visual Format, and Health Literacy on information recall (*F*_1,223_=4.22, *P*=.04, η_p_^2^=.02). As shown in Table 3, this interaction suggests that, in the case of spoken texts, animations result in higher recall scores among people with low health literacy compared to illustrations. This effect was not found in people with high health literacy, confirming H4b.

Our fifth hypothesis concerned the influence of animated visuals on attitudes toward the message, in the case of spoken messages. No interaction was found between Text Modality and Visual Format on attitudes toward the message (*F*_1,223_=0.14, *P*=.71, η_p_^2^=.001). This was not expected and H5 was, therefore, rejected.

**Figure 3:**
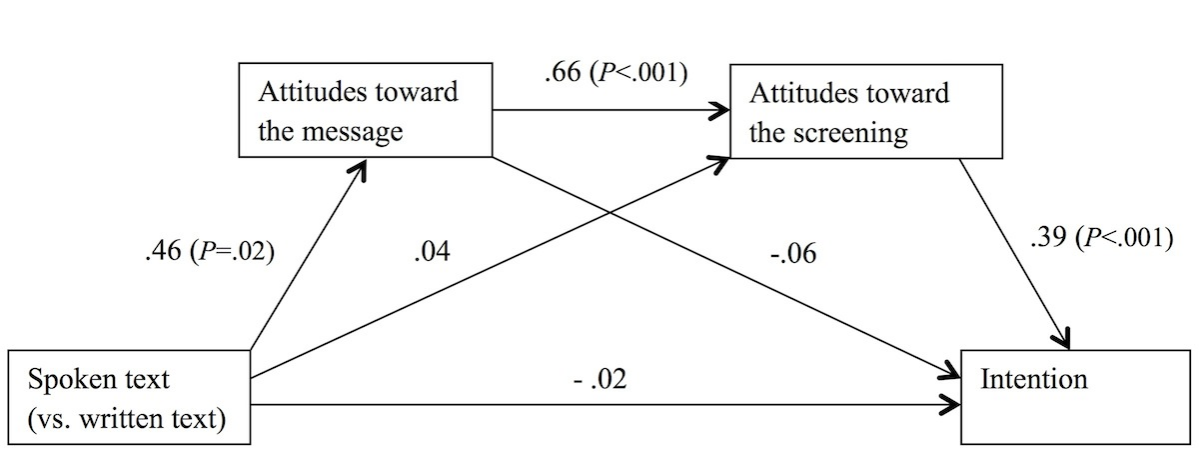
Influence of text modality on intention to screen, mediated by attitudes toward the message and attitudes toward the screening (n=108). Unstandardized regression coefficients are presented.

Mediation analysis showed a significant indirect effect of spoken text (controlling for visual format) on the intention to screen (*b*=.12, 95% CI 0.02-0.25) in people with low health literacy. Compared to written texts, spoken messages positively affected people’s attitudes toward the message. This, in turn, influenced attitudes toward the screening, which improved screening intention. Figure 3 shows the mediation model with the direct effects (unstandardized coefficients).

The indirect effects of the serial mediation model are presented in Table 4. The results show that spoken text positively affected the intention to screen, but only through attitudes toward the message and attitudes toward the behavior. The indirect effects of the single mediator models are not significant on a 95% confidence level, indicating that both mediators contribute to the effect. With this finding, the sixth hypothesis is supported.

**Table 2:** Main effects of text modality on information recall and attitudes toward the message in people with low and high health literacy.^a^

Group		n	Information recall Scale range: 0-28		Attitudes toward the message Scale range: 1-7	
			mean (SE)	95% CI	mean (SE)	95% CI
**All participants**						
	Written text	126	11.97^b^(0.46)	11.06-12.89	5.79^c^(0.09)	5.62-5.96
	Spoken text	105	13.60 (0.52)	12.58-14.61	6.15 (0.10)	5.97-6.35
**Low health literacy**						
	Written text	64	9.12^d^(0.66)	7.83-10.41	5.75^e^(0.12)	5.51-5.99
	Spoken text	44	11.42 (0.79)	9.87-12.98	6.20 (0.15)	5.91-6.49
**High health literacy**						
	Written text	62	14.83 (0.66)	13.51-16.14	5.83 (0.12)	5.59-6.07
	Spoken text	61	15.77 (0.67)	14.45-17.09	6.11 (0.13)	5.86-6.35

^a^Higher scores indicate more recall and positive attitudes.

^b^Differs significantly from spoken text in all participants (*P*=.02).

^c^Differs significantly from spoken text in all participants (*P*=.01).

^d^Differs significantly from spoken text in low health literacy group (*P*=.03).

^e^Differs significantly from spoken text in low health literacy group (*P*=.02).

**Table 3:** Interaction effects of text modality and visual animation in people with low or high health literacy.^a^

Group	n	Information recall Scale range: 0-28		Attitudes toward the message Scale range: 1-7	
		mean (SE)	95% CI	mean (SE)	95% CI
Low - written - illustration	29	9.59 (0.97)	7.67-11.50	5.78 (0.18)	5.42-6.13
Low - written - animation	35	8.66 (0.88)	6.92-10.40	5.71 (0.17)	5.39-6.04
Low - spoken - illustration	23	9.61^b^(1.08)	7.47-11.75	6.22 (0.20)	5.82-6.62
Low - spoken - animation	21	13.24 (1.14)	11.00-15.48	6.19 (0.21)	5.77-6.60
High - written - illustration	33	14.52 (0.91)	12.73-16.30	5.87 (0.17)	5.53-6.20
High - written - animation	29	15.14 (0.97)	13.23-17.05	5.80 (0.18)	5.44-6.15
High - spoken - illustration	29	16.03 (0.97)	14.13-17.94	6.03 (0.18)	5.67-6.39
High - spoken - animation	32	15.50 (0.92)	13.68-17.32	6.18 (0.17)	5.84-6.52

^a^Higher scores indicate more information recalled and positive attitudes.

^b^Mean differs significantly when comparing low health literates in the spoken animation condition to those in the spoken illustration condition (*P*=.02).

**Table 4:** Total and indirect effects for text modality on intention mediated by attitudes toward the message and attitudes toward the screening (n=108).

Indirect effect	Estimate (SE)	Bootstrap 95% CI
Total	0.11 (0.07)	−0.03 to 0.25
modality → attitude to message → intention	−0.03 (0.03)	−0.10 to 0.01
modality → attitude to message → attitude to screening → intention	0.12 (0.06)	0.02 to 0.25
modality → attitude to screening → intention	0.01 (0.06)	−0.10 to 0.13

## Discussion

### Principal Findings

This study investigated the effectiveness of animated features among people with either low or high health literacy. Six hypotheses were tested, with four being confirmed and one being partly confirmed. The results showed that spoken messages are better recalled and induce more positive attitudes compared to written texts (H1 and H2). Animated messages with spoken text result in more recall and positive attitudes compared to illustrations. Both effects applied only to low health literates (H3 and H4b). In the low health literate group, message format indirectly influenced intention to get cancer screening through both attitudes toward the message and attitudes toward the screening (H6). Animations did not significantly improve people’s attitudes toward the message, rejecting H5. Textbox 1 provides an overview of the hypotheses and findings.

**Overview of the hypotheses and findings of the study.**

- H1: Health messages with spoken text (vs written text) improve information recall.
  - Supported. Spoken messages were significantly better recalled than written messages, indicated by a main effect for text modality on information recall.
- H2: Health messages with spoken text (vs written text) result in positive attitudes to the message.
  - Supported. Spoken messages resulted in significantly more positive attitudes toward the message compared to written messages, indicated by a main effect for text modality on attitudes toward the message.
- H3: The positive effect of spoken text (vs written text) on recall and attitude to the message exists only among people with low health literacy.
  - Supported. Simple effect analysis showed that spoken text (compared to written text) only improved recall and attitudes to the message in the low health literacy group, not for people with high health literacy.
- H4a: Animations (vs illustrations) positively affect recall, but only if the text is spoken.
  - Not supported. Overall, no interaction was found between text modality and type of visualization on information recall.
- H4b: This interaction effect will only exist among people with low health literacy.
  - Supported. A significant three-way interaction was found showing that in the case of spoken messages, animations (compared to illustrations) result in higher recall scores among people with low health literacy. This effect was not found in people with high health literacy.
- H5: Animations (vs illustrations) positively influence attitudes to the message, but only if the text is spoken.
  - Not supported. No interaction was found between text modality and type of visualization on attitudes toward the message.
- H6: Among people with low health literacy, spoken text (vs written text) improves the intention to screen for cancer. This relationship is mediated by both the attitude toward the message and the attitude toward the behavior.
  - Supported. Spoken text indirectly improved intention to have screening in people with low health literacy. Compared to written texts, spoken messages positively affected people’s attitudes toward the message, which influenced screening attitude and subsequently screening intention.

The results of our study support the modality effect that is part of the cognitive theory of multimedia learning [14]. In addition to the students who often participate in modality experiments, this study shows that vulnerable groups in society—those having low health literacy—learn better from multimodal information as well. Although people with low health literacy especially seem to benefit from animated health messages, our study also showed that animated messages do not induce negative effects among people with high health literacy. This is in line with a study on tailored health information, which showed that audiovisual messages on smoking cessation are effective, regardless of education level [6]. This study adds to the literature by focusing on the specific features of animations that influence information processing in different health literacy groups. By doing this, the effective components of either audiovisual messages or written text messages could be identified, providing better insight into the usefulness of animations in reducing disparities in health information processing.

The effectiveness of animations in health communication likely depends on the type of content that is presented. Our messages described the development of colorectal cancer, how bowel polyps are removed, and the testing procedure, which can be easily shown in an animation. Other types of content are most likely less easily visualized. It is possible that the positive effect of animations therefore does not apply to informed consent information, for example, which would explain the negative result in one of the studies [42]. The studies that found positive results for audiovisual messages focused on sleep apnea and the functionality of positive airway pressure [43], or inhaler use in asthma [44]. It can therefore be expected that animations are effective when the images truly represent the content of the message and contribute to its understanding. If this is not the case, the movement of animations could potentially distract from the content. In that case, people exposed to animations could primarily remember the fact that they saw an animation instead of its content [28]. In our study, the animation clearly represented the text without adding additional and possibly distracting content, which could explain our findings.

Different explanations apply to the finding that spoken information is better recalled by people with low health literacy. It is possible that information through multiple modes improves information processing, as predicted by the cognitive theory of multimedia learning. Another explanation relates to the fact that health literacy and functional literacy are associated [21]. Possibly, participants with low health literacy were less skilled readers, which might have caused the superiority of the spoken messages where no reading was required. Although we controlled for the influence of education level in this study by stratifying our sample, we did not test actual reading ability. Future research should, therefore, disentangle the mechanism underlying this finding.

### Limitations

A limitation of this study relates to the experimental messages of this study. We divided the messages into 15 short segments that could be replayed. We intentionally provided participants with the opportunity to replay the messages to avoid pacing differences between the spoken and written conditions [22]. However, tracking data of the participants’ clicking behavior revealed that only a few participants actually made use of this opportunity. A disadvantage of the split-up into shorter segments is that the animation was not as natural as possible. In a natural setting, animations can be viewed entirely and not as separate pieces. Future research should, therefore, address modality differences and animations in longer messages. However, it could be expected that complete animations are even better processed because the exposure is more fluent and not disturbed by unnatural stops. Moreover, a recent meta-analysis on the modality effect has shown that the superiority of spoken messages over written messages has mainly been found in system-paced messages [27]. The fact that our study showed a modality difference in self-paced messages adds to the expectation that for longer, system-paced messages, modality differences will be even larger.

In our study, we aimed to identify the specific message features that impact the way in which people with different health literacy levels process information. We used the SAHL-D as an indicator of health literacy as it objectively measures comprehension of health-related information. However, to successfully make use of the information that is available online, people need multiple skills. For example, finding relevant information online and judging the information for its credibility goes beyond our health literacy measure. These skills are better captured by an eHealth literacy scale such as the eHEALS [45]. Including eHealth literacy in future research on information on colorectal cancer screening might be relevant, as eHealth literacy has shown to be related to colorectal cancer knowledge and screening participation [46]. A disadvantage of the eHEALS measure is, however, that it does not always adequately reflect people’s actual performance on online tasks [47]. As our study addressed the influence of health literacy on quality of information processing, we considered SAHL-D to be the best health literacy measure for this purpose, also in an online setting.

### Conclusions

To conclude, the findings of this study show that animated visual information combined with spoken text is the best way to communicate complex health messages to people with low health literacy. This format can even bridge the gap between audiences with low and high health literacy as the recall differences between the two groups are eliminated. Spoken information generates more positive attitudes toward the message, as well as the screening, and improves the intention to screen in people with low health literacy. It must be noted that the animations and narrated text were both of professional quality. The animations were made by a professional animator and the text was narrated by a professional radio news presenter. This could also have induced positive attitudes toward the message. There are free or inexpensive programs available to make animations. However, the limited options of these programs might not be sufficient to make a good, credible, and professional-looking animation. Future research should investigate whether the design quality of animations actually influences message effects. For now, we recommend the use of professional software packages when designing health animations. In this study, spoken animations improved information processing among people with low health literacy, whereas no negative format effects were observed in people with high health literacy. This conclusion indicates that, in public health messages, information adapted to audiences with low health literacy suits people with high health literacy as well.

## Multimedia Appendix 1

The entire animated message combined with spoken text.

## Abbreviations

eHEALS: eHealth Literacy Scale
SAHL-D: Short Assessment of Health Literacy in Dutch
